# Nomogram for predicting testicular yolk sac tumor in children based on age, alpha-fetoprotein, and ultrasonography

**DOI:** 10.3389/fped.2024.1407120

**Published:** 2024-11-13

**Authors:** Huan Yu, Hui Wang, Yichen Huang, Huiyong Hu, Yue Zhang, Min Wu, Yiqing Lyu, Yan Chen, Lijun Zhou, Yan Liang, Huizhen Sun, Xiaoling Lin, Hua Xie, Fang Chen

**Affiliations:** ^1^ Department of Urology, Shanghai Children's Hospital, School of Medicine, Shanghai Jiao Tong University, Shanghai, China; ^2^ Department of Ultrasound, Shanghai Children's Hospital, School of Medicine, Shanghai Jiao Tong University, Shanghai, China; ^3^ School of Life Sciences and Biotechnology, Shanghai Jiao Tong University, Shanghai, China

**Keywords:** testicular tumor, AFP, ultrasonography, children, nomogram

## Abstract

**Objective:**

To establish a predictive model for distinguishing testicular benign or yolk sac tumors in children.

**Methods:**

We retrospectively analyzed data for 119 consecutive patients with unilateral testicular tumors treated at a single institution from June 2014 to July 2020. The patients were divided into the benign (*n* = 90) and yolk sac (*n* = 29) tumor groups based on the pathological diagnosis. We recorded patient age, serum markers [serum alpha-fetoprotein (AFP), human chorionic gonadotropin], and tumor ultrasonic findings (maximum diameter, ultrasonic echo, blood flow signal). Predictive factors were identified using descriptive statistical methods. A nomogram was established for preoperative prediction. An additional 46 patients were used as a validation cohort to verify the model.

**Results:**

Patients with testicular yolk sac tumors were younger (median age: 14.0 vs. 34.0 months, *P* = 0.001) and had a higher incidence of elevated AFP levels (93.1% vs. 2.2%, *P* < 0.001). Ultrasonography indicated that testicular yolk sac tumors tended to have larger maximum diameters (26.5 ± 11.3 vs. 16.6 ± 9.2 cm, *P* < 0.001), a higher proportion of hypoechoic masses (44.8% vs. 8.9%, *P* < 0.001), and a higher incidence of masses with strong blood flow signals (93.1% vs. 5.6%, *P* < 0.001). A nomogram based on age, AFP levels, and ultrasound blood flow signals effectively predicted the probability of yolk sac tumor in children, with an accuracy of 0.98 (95% confidence interval: 0.984–1.003). The Brier score of the nomogram was 0.0002.

**Conclusion:**

A nomogram based on age, AFP levels, and ultrasound blood flow signals can effectively predict the probability of testicular yolk sac tumor preoperatively, aiding in clinical decision-making and patient counseling.

## 1 Introduction

Testicular tumors in prepubertal children are rare, with an incidence of 0.5–2 per 100,000 children (1). The surgical treatment approach of testicular tumors in prepubertal children is different from that in postpubertal adolescents and adults (2). For benign tumors, such as mature teratomas, testicular-sparing surgery (TSS), which preserves testicular tissue, is typically performed (2–4). However, for the most common malignant testicular tumor in children, yolk sac tumor, a radical orchiectomy is required. Therefore, distinguishing between testicular benign or yolk sac tumors before surgery is crucial. Currently, the most commonly used preoperative diagnostic methods include serum tumor markers, ultrasonography, and magnetic resonance imaging (MRI). Alpha-fetoprotein (AFP) is the most widely used serum biomarker for identifying testicular yolk sac tumors in children. However, AFP levels can be elevated in infants and other diseases, and some patients with yolk sac tumors may even present with normal AFP levels. While ultrasonography is a preferred imaging option, it has relatively low specificity and is somewhat subjective. Therefore, it is advantageous to combine multiple clinical indicators to better distinguish between benign and yolk sac tumors. In this study, we analyzed the correlation between preoperative parameters and pathological outcomes in pediatric patients with testicular tumors, identifying predictors of yolk sac tumors and establishing a predictive model using a nomogram.

## 2 Materials and methods

### 2.1 Clinical data

All records of 119 testicular tumor patients from our hospital from June 2014 to July 2020 were identified in this study. This study was approved by the Ethics Review Committee of Shanghai Children's Hospital, Shanghai Jiao Tong University (Shanghai, China) (Approval No.: 2022R024-E01). Informed consent forms were not required. The inclusion criteria were: (1) prepubertal boy, (2) primary unilateral testicular tumor, and (3) relatively complete preoperative data, while the exclusion criteria were (1) bilateral testicular or metastatic tumors (2) history of cryptorchidism, family history of testicular tumors, or genetic syndrome. All patients were categorized into testicular benign and yolk sac tumor groups based on the pathological diagnosis, following the 2016 World Health Organization classification of testicular tumors (5). A senior pathologist reviewed the pathological diagnoses for all children. The data collected included the patient's age, clinical findings, serum tumor markers [including AFP and human chorionic gonadotropin (HCG)], and Doppler ultrasound findings of the testicular tumors (maximum diameter, echogenicity, and blood flow signals). The reference range for the normal value of AFP was based on previous research findings (6). The Adler semiquantitative classification was applied to assess blood flow, categorizing it into four grades (7): Grade 0 indicates no detectable blood flow signals; Grade I refers to 1 or 2 small blood vessels with a diameter of <1 mm; Grade II refers to 3 or 4 small blood vessels; and Grade III indicates more than 4 blood vessels or vessels that are interconnected and form a network. Ultrasound images of all enrolled children were reviewed by a sonographer and a urologist.

### 2.2 Statistical analyses

Continuous variables were compared using the Mann-Whitney *U* test, while categorical variables were analyzed with *χ*² tests. Multivariable logistic regression was employed to evaluate predictive factors for testicular benign and yolk sac tumors in children. The diagnostic performance of these predictive factors in differentiating between testicular benign and yolk sac tumors was assessed using receiver operating characteristic (ROC) curve analysis. A nomogram was developed using R software (The R Foundation for Statistical Computing, Vienna, Austria). All other statistical analyses were conducted using SPSS version 24.0 (IBM Corp., Armonk, NY, USA). A *P*-value of <0.05 was considered statistically significant.

## 3 Results

### 3.1 Pathological diagnosis

Among the 119 cases, 90 were benign tumors. These included 82 teratomas (68.3%), of which 41 were mature teratomas, 5 were immature teratomas, and 36 were epidermoid/dermoid cysts. Additionally, there were six Leydig cell tumors (5%), one capillary hemangioma, and one borderline serous papillary cystadenoma. The remaining 29 cases were yolk sac tumors (24.4%), including one case which presented as yolk sac tumors with teratoma.

### 3.2 Predictive factors

The median age of the children was 25.0 months (1.7–160 months). The 29 patients (24.4%) with testicular yolk sac tumors were significantly younger than another 90 patients (75%) with benign testicular tumors (14.0 vs. 34.0 months, *P* = 0.001). The study population consisted of 40 patients (33.6%) under 1 year old, 39 patients (32.8%) aged 1–3 years, 11 patients (9.2%) aged 3–5 years, and 30 patients (24.4%) older than 5 years.

Regarding the serum tumor markers, preoperative serum HCG levels were within the normal range in all children (0–5 mIU/ml). However, elevated serum AFP was noticed in 27/29 cases (93.1%) for the yolk sac tumor group while the incidence was 2.2% (2/90) in the benign group (*P* < 0.001).

The maximum diameter of the testicular yolk sac tumor determined by ultrasound was significantly larger than that of the benign tumors (26.5 ± 11.3 vs. 16.6 ± 9.2 mm, *P* < 0.001). Yolk sac tumor also tended to be hypoechoic echo (44.8% vs. 8.9%, *P* < 0.001), and showed Grade III blood flow signals (93.1% vs. 5.6%, *P* < 0.001) (Table 1).

**Table 1:** Distribution of clinical characteristics in 119 children with testicular tumors.

Variables	All patients (*n* = 119)	Benign testicular tumor cases (*n* = 90)	Testicular yolk sac tumor cases (*n* = 29)	*P*-value
Age (months)				0.001*
Mean ± s.d.	44.3 ± 45.9	53.0 ± 49.6	17.3 ± 8.1	
Median (range)	25.0 (1.7–160.0)	34.0 (1.7–160.0)	14.0 (4.7–35.0)	
AFP, *n* (%)				<0.001*
Normal range	86 (72.3)	84 (93.3)	2 (6.9)	
Elevated value	29 (24.4)	2 (2.2)	27 (93.1)	
Data unavailable	4 (3.4)	4 (4.5)	0 (0)	
Tumor diameter (mm)				<0.001*
Mean ± s.d.	19.0 ± 10.6	16.6 ± 9.2	26.5 ± 11.3	
Median (range)	18.0 (3.0–58.0)	14.2 (3.0–55.0)	25.0 (10.0–58.0)	
Data unavailable	8 (6.7)	6 (6.7)	2 (6.7)	
Ultrasound echo, *n* (%)				<0.001*
Echoless	17 (14.3)	17 (18.9)	0 (0)	
Hypoechoic	21 (17.6)	8 (8.9)	13 (44.8)	
Isoechoic	7 (5.9)	0 (0)	7 (24.1)	
Heterogeneous echo	69 (58.0)	61 (67.8)	8 (27.6)	
Data unavailable	5 (4.2)	4 (4.4)	1 (3.4)	
Ultrasound blood flow, *n* (%)				<0.001*
Grade 0	48 (40.3)	47 (52.2)	1 (3.4)	
Grade I	20 (16.8)	20 (22.2)	0 (0)	
Grade II	5 (4.2)	5 (5.6)	0 (0)	
Grade III	32 (26.9)	5 (5.6)	27 (93.1)	
Data unavailable	14(11.8)	13(14.4)	1(3.4)	

AFP, alpha-fetoprotein; s.d., standard deviation.

* *P* < 0.05.

The patient's age, serum AFP levels, maximum tumor diameter, ultrasonic echo, and blood flow signal were identified as predictive factors for differentiating between testicular benign and yolk sac tumors (Table 1).

### 3.3 Contribution and validation of the nomogram

**Figure 1:**
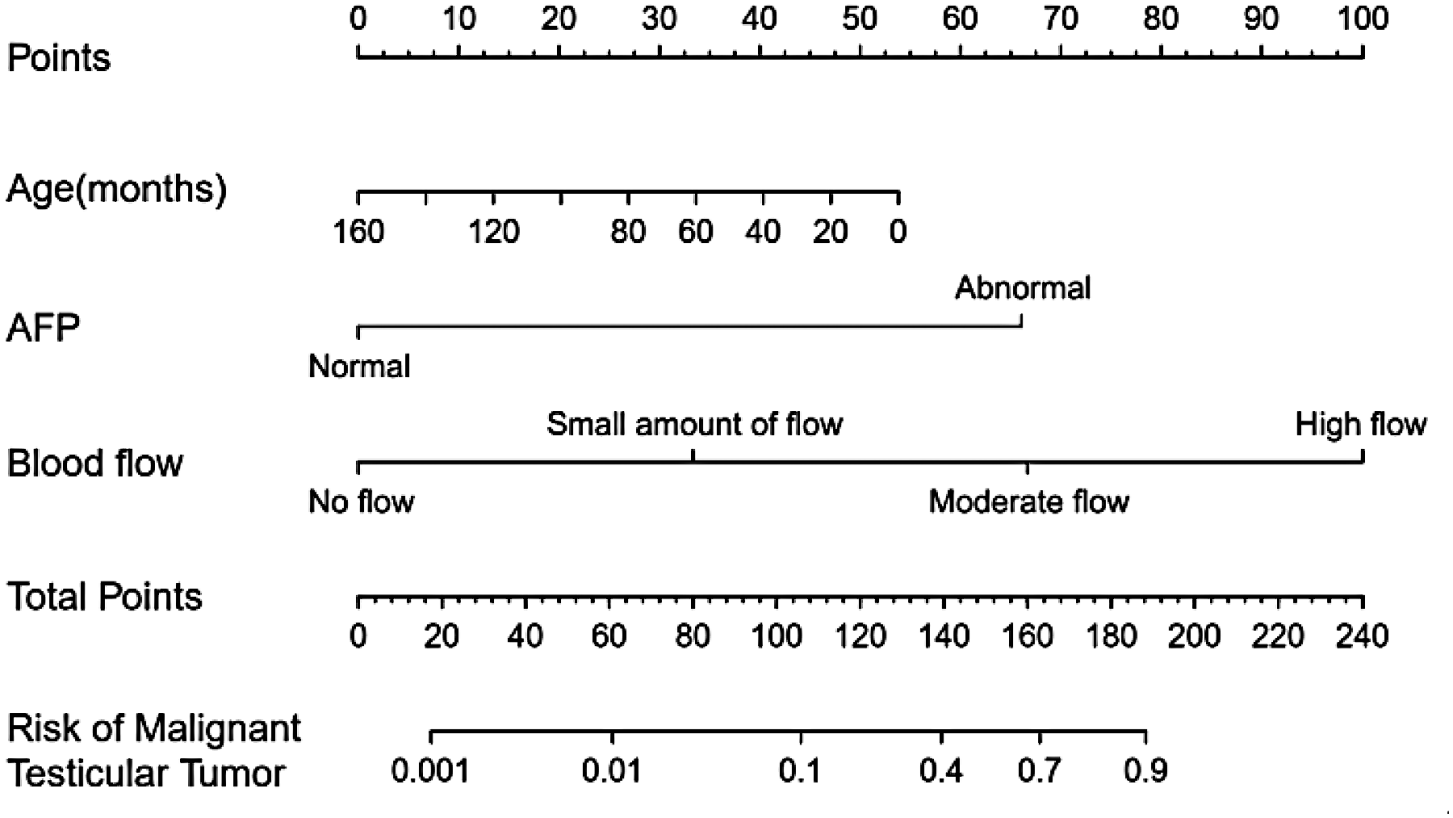
Nomogram for predicting the probability of testicular yolk sac tumor in children.

Multivariable logistic regression analysis was performed to generate a ROC curve, which resulted in an area under the curve (AUC) of 0.984, with a sensitivity of 0.978 and a specificity of 0.966 (Table 2, Supplementary Figure 1). AFP (*P* = 0.031) and ultrasound blood flow signals (*P* = 0.001) were identified as significant predictors of testicular yolk sac tumors in children. To construct the optimal predictive model (nomogram) for malignancy probability, Harrell's concordance index (C-index) was used, incorporating age, AFP levels, and ultrasound blood flow signals (Figure 1). The calibration plot (Supplementary Figure 2) demonstrated an average absolute error of 0.028 between the malignancy probability predicted by the nomogram and the actual incidence of malignancy in the study population. The predictive accuracy of the nomogram model was 0.98 (95% confidence interval: 0.984–1.003).

**Table 2:** Predictive factors for testicular yolk sac tumor in children.

Variable	OR (95%CI)	*P*-value
Age (months)	0.973 (0.929–1.02)	0.256
AFP	1.007 (1.001–1.013)	0.031*
Tumor diameter	0.945 (0.8831–1.097)	0.976
Ultrasound blood flow	6.916 (2.116–22.604)	0.001*

CI, confidence interval.

* *P* < 0.05.

The left side of the nomogram (Figure 1) included three key markers. The first step in using the nomogram involved determining the corresponding points for each marker in the top row based on the patient's data. After obtaining these points, the next step was to calculate the total score by summing the points from each marker, which then allowed for the estimation of the risk of a testicular yolk sac tumor (last row). For example, in a 10-month-old male child who presented with a painless scrotal mass, an AFP level of 23,154 ng/ml, and Grade III blood flow signals on ultrasound, the total score would be approximately 215 points (50 + 65 + 100). This score corresponded to an estimated probability of nearly 100% for a testicular yolk sac tumor.

**Figure 2:**
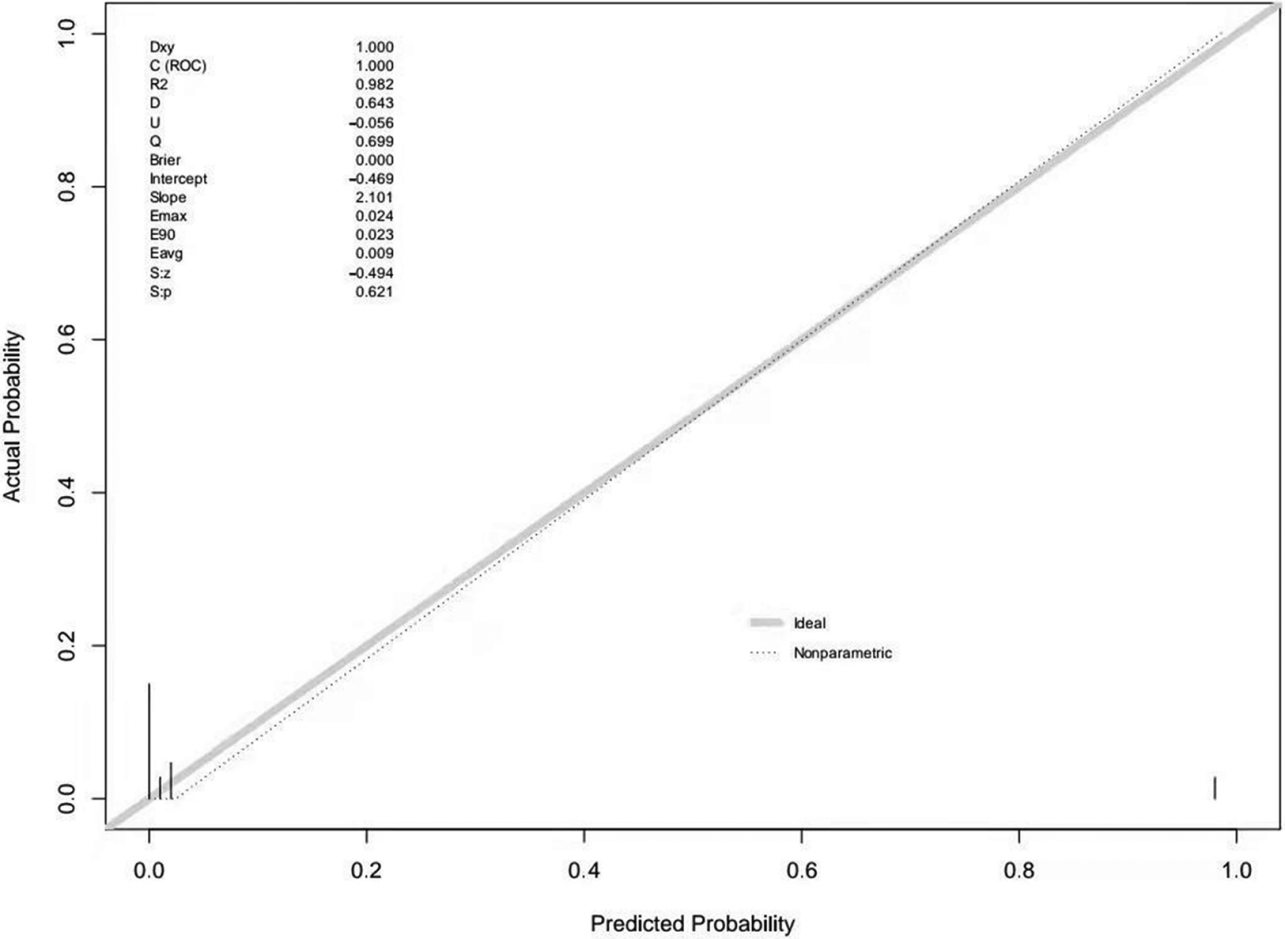
The brier score for the prediction nomogram.

The external validation of the nomogram was performed using data from 46 children with testicular tumors admitted to our hospital from July 2021 to August 2023. The Brier score of the nomogram was 0.0002, which was close to 0, indicating great predictive ability (Figure 2). The corrected C-index generated by external validation was 1.

## 4 Discussion

Prepubertal testicular tumors account for 1%–2% of pediatric solid tumors (1) and are classified into germ cell and non-germ cell tumors based on their origin. Germ cell tumors, which include yolk sac tumors and teratomas (mature/immature teratomas, dermoid cysts, epidermoid cysts), are the predominant type, comprising 95% of all adult and prepubertal testicular tumors (5). In contrast, non-germ cell tumors consist of sex cord-stromal tumors and metastatic tumors. In our study, 93.3% of the tumors were germ cell tumors. The prevalence of teratomas and yolk sac tumors varies across different studies, potentially due to racial differences or significant selection biases that may lead to the underreporting of benign tumors. Historically, it was believed that more than 60% of germ cell tumors were yolk sac tumors, with teratomas accounting for approximately 25% (2). However, a recent single-center study in Japan, which included 62 cases (8), reported that the proportions of yolk sac tumors and teratomas were 42% and 47%, respectively. In contrast, a multicenter study in North America found that teratomas and yolk sac tumors occurred in 62% and 15% of cases, respectively (9). These findings are consistent with our study, which observed 68.9% teratomas and 24.4% yolk sac tumors.

Testicular tumors tend to occur in two distinct age periods (10). The first is before the age of 3, and the second is from puberty to the fourth decade of life, during which testicular tumors are the most common type of tumors in men. In our study, 66.4% of patients were under 3 years old. Notably, all 29 cases of yolk sac tumors in our study were in children younger than 3 years old, with a median age of 14 months (4.7–35 months). This age-related pattern is consistent with findings by Cornejo et al. (11), who reported a median age of 17 months (5–71 months) among 33 children with yolk sac tumors. For benign tumors, Karmazyn et al. (12) found that all benign testicular tumors occurred in children aged 5–12 years. Similarly, in our study, all 30 cases in children older than 5 years were benign tumors. These findings suggest that the incidence of testicular yolk sac tumor decreases with increasing age in this patient population.

TSS is commonly used to treat benign testicular tumors in children, while testicular yolk sac tumor is typically managed with radical orchiectomy. Therefore, accurately differentiating between benign and yolk sac tumor preoperatively is crucial for selecting the appropriate treatment approach. Although there have been a few comprehensive studies on the differential diagnosis of testicular benign and yolk sac tumors in children, most have focused on evaluating the diagnostic value of specific methods. For instance, Ricardo et al. (13) highlighted the roles of various testicular tumor markers, such as miR-367-3p, miR-371a-3p, and circulating tumor DNA, which were found to be more sensitive than AFP and HCG. Additionally, Markus et al. (14) suggested using contrast-enhanced ultrasound for the differential diagnosis of testicular tumors, achieving a sensitivity of 93% and a specificity of 94%. However, these advanced methods have yet to see widespread clinical application.

Serum AFP is a crucial marker for diagnosing yolk sac tumors in children. Typically, AFP levels remain within the normal range in prepubertal testicular teratomas, though they may occasionally be elevated in cases involving large tumors (15) or very young children. AFP levels are significantly elevated in approximately 90% of patients with yolk sac tumors (16), meaning that in about 10% of these patients, AFP levels do not increase. In children younger than 1 year with testicular tumors, an elevated AFP level may also be observed in those with benign tumors. Conversely, in children older than 1 year, a normal AFP level often suggests a benign tumor. In our study, the two patients with elevated AFP levels were 4 months and 8 months old, suggesting that their elevated AFP levels could be related to their age rather than the malignancy of the tumor. HCG, on the other hand, appears to have limited diagnostic value for prepubertal testicular tumors, as all cases in our study exhibited normal HCG levels. Additionally, in our study, three yolk sac tumors presented with normal AFP levels, underscoring the limitations of relying solely on AFP as a predictor for testicular yolk sac tumor. This finding is consistent with previously reported data (16).

Ultrasonography is considered the primary imaging modality for evaluating testicular tumors, as it effectively assesses the tumor's size, internal blood flow signals, and echogenicity. Yolk sac tumors can present with various sonographic appearances, including as focal homogeneous lesions, a solid mass with cystic areas or components, or as heterogeneous diffuse lesions (17). In our study, the maximum diameter of the testicular yolk sac tumors were larger than that of benign tumors, a finding consistent with previous multicenter studies (18). However, a tumor diameter cutoff of 2 cm alone was not sufficient to accurately predict the pathological diagnosis in children with testicular tumors (19). Our study also suggested that the ultrasound echogenicity of benign testicular tumors may be higher than that of yolk sac tumors, with a higher proportion of yolk sac tumors appearing hypoechoic or isoechoic. However, this assessment is highly dependent on the subjective judgment of the sonographer. Due to the rich vascular supply characteristic of testicular yolk sac tumors, high blood flow signals detected by ultrasound can be a useful indicator for distinguishing testicular yolk sac tumors, as confirmed by our findings.

A nomogram is a predictive model for disease occurrence and progression that is based on multivariate regression analysis. It simplifies complex calculation formulas into an easy-to-use scoring system by employing geometric figures to represent mathematical regression models, visually displaying the interaction or cumulative effects among the predictive indicators. As a result, nomograms have become widely used in clinical research as multiparameter prediction models, including for the prognosis of germ cell tumors across all age groups (20), as well as for conditions like prostate cancer (21), lung cancer (22), and Crohn's disease (23). In our study, univariate analysis identified age, maximum tumor diameter, ultrasound echogenicity, and ultrasound blood flow as potential indicators for distinguishing between benign and testicular yolk sac tumors in children. We then developed a nomogram using multivariate analysis, which highlighted age, AFP levels, and ultrasound blood flow signals as key predictive factors. This model demonstrated a high predictive accuracy for testicular yolk sac tumors in children, with an accuracy rate of 0.98. To our knowledge, this is the first nomogram model designed specifically to predict prepubertal testicular tumors. The nomogram achieved a Brier score of 0.0002, indicating excellent predictive ability. Further validation confirmed that this nomogram has strong predictive accuracy and significant potential for clinical application. This nomogram can be used for preoperative evaluation and to provide clear explanations to parents.

However, there were limitations in this study. As a retrospective study, the nomogram model we developed lacked sufficient external validation. Additionally, the limited number of cases in our dataset prevented us from establishing a model capable of predicting specific pathological outcomes. It's important to note that no current diagnostic tool is 100% sensitive or specific. The main utility of the nomogram would be to prepare for the approach and provide proper counseling to the family about the likelihood of finding a testicular yolk sac tumor. Although MRI has shown promise for the diagnosis of testicular tumors (24), it is not yet routinely employed in all cases due to its high cost and the lack of standardized protocols.

## 5 Conclusion

Age, serum AFP levels, maximum tumor diameter, and ultrasound blood flow signals are key predictive factors for testicular yolk sac tumors in children. A nomogram incorporating these factors can effectively predict the probability of testicular yolk sac tumor preoperatively, aiding in clinical decision-making and patient counseling.

## Data Availability

The original contributions presented in the study are included in the article/Supplementary Material, further inquiries can be directed to the corresponding author.
