# Supplementary material for: Nomogram for predicting testicular yolk sac tumor in children based on age, alpha-fetoprotein, and ultrasonography
Source: Front Pediatr. 2024 Nov 13;12:1407120. doi: 10.3389/fped.2024.1407120 (PMC11598321; doi:10.3389/fped.2024.1407120)
Supplement: Supplementary file 1 [file Datasheet1.pdf]

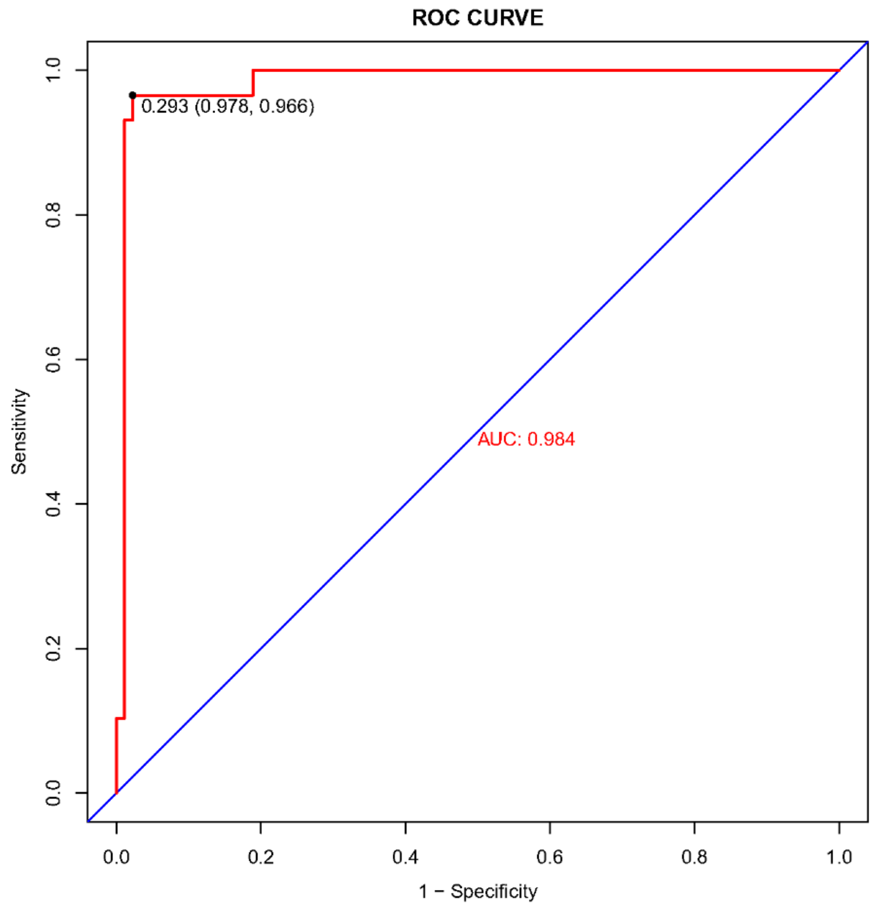

Supplementary Figure 1: Receiver operating characteristic curve for diagnosing testicular yolk sac tumor

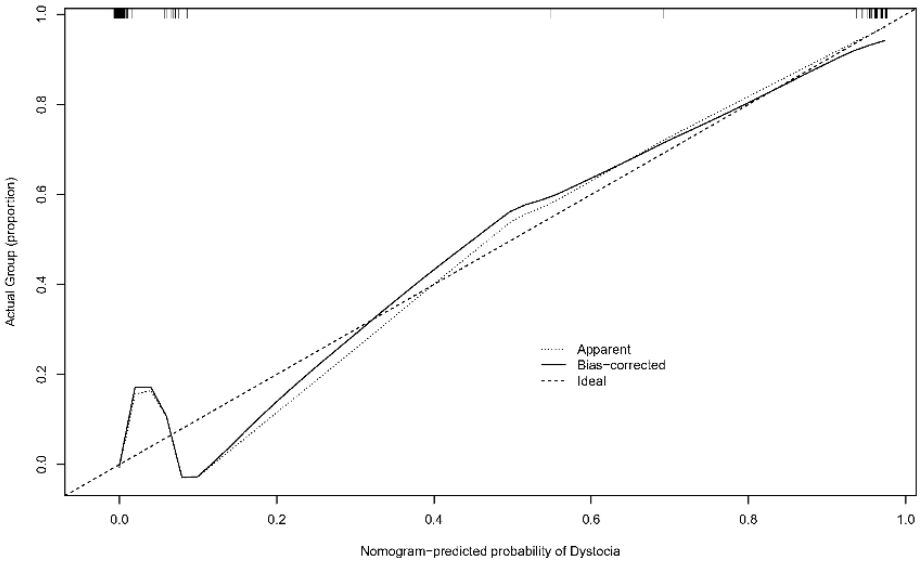

Supplementary Figure 2: Calibration plot for predicting the probability of testicular yolk sac tumor
